# Supplementary material for: Children’s Particulate Matter Exposure Characterization as Part of the New Hampshire Birth Cohort Study
Source: Int J Environ Res Public Health. 2021 Nov 18;18(22):12109. doi: 10.3390/ijerph182212109 (PMC8620988; doi:10.3390/ijerph182212109)

Supplemental Material

Tables S1 and S2 present cohort demographics and home heating and tobacco exposure survey results, respectively, for the 258 children included in this analysis. Nearly 23% of enrolled children live in a home with a wood stove as the primary heating source.

Table S1. Selected Parent and Child Characteristics

		Mean (SD)
Maternal education level (%)	Any post-graduate schooling	75 (29.1)
	College graduate	103 (39.9)
	High school graduate or equivalent	17 (6.6)
	Junior college graduate, or some college or technical school	42 (16.3)
	Less than 11th grade	1 (0.4)
	Unknown	20 (7.8)
Paternal education level (%)	Any post-graduate schooling	40 (15.5)
	College graduate	82 (31.8)
	High school graduate or equivalent	53 (20.5)
	Junior college graduate, or some college or technical school	57 (22.1)
	Less than 11th grade	2 (0.8)
	Unknown	24 (9.3)
Child's sex (%)	Female	118 (45.7)
	Male	140 (54.3)
Collection season (%)	Fall	64 (24.8)
	Spring	63 (24.4)
	Summer	70 (27.1)
	Winter	61 (23.6)
Maternal age at study enrollment, yrs		32.1 (4.9)
Child's age in months		46.0 (2.5)
Child's BMI		16.2 (1.5)
Child's BMI-for-age Z-score		0.46 (1.15)
Child sleep duration, hrs		32.77 (7.43)
Percentage of time child was estimated to sleep during the collection period,		0.44 (0.08)

Table S2. Home Heating and Tobacco Smoke Exposure at the time sampling began.

		Mean (SD)
Primary home heating appliances (%)	Fireplace	1 (0.4)
	Forced air (furnace)	106 (41.1)
	Other	14 (5.4)
	Pellet stove	15 (5.8)
	Radiators (boiler)	60 (23.3)
	Unknown	4 (1.6)
	Wood stove	58 (22.5)
Primary home heating fuel (%)	Electricity	9 (3.5)
	Kerosene	1 (0.4)
	Natural Gas	5 (1.9)
	Oil	91 (35.3)

	Other	1 (0.4)
	Propane	69 (26.7)
	Wood	62 (24.0)
	Wood pellets	16 (6.2)
	Unknown	2 (0.8)
Exposure to tobacco smoke (yes/no) (%)	Yes	29 (11.2)
	No	225 (87.2)
	Unknown	2 (0.8)
Number of air filters at home (%)	None	227 (88.0)
	1 or more	31 (12.0)
Fireplace exposure (yes/no) (%)	Yes	4 (1.6)
	No	253 (98.1)
Woodstove exposure (yes/no) (%)	Yes	51 (19.8)
	No	205 (79.5)
	Unknown	1 (0.4)
Duration of fireplace exposure, hrs (mean (SD))		0.11 (1.51)
Duration of woodstove exposure, hrs (mean (SD))		2.39 (6.07)
Duration of tobacco smoke exposure, hrs (mean (SD))		0.13 (0.57)

Table S3A. Exposure levels according to selected characteristics of the study population from univariate analyses.

	N (%)	$PM_{2.5}$ GM	$PM_{2.5}$ GSD	P-value*	BrC GM	BrC GSD	P-value*	BC GM	BC GSD	P-value*
**Overall**	174	11.2	2.0		3.8	2.1		2.5	2.2	
**Maternal education level (%)**				>0.05			<0.01			<0.05
Any post-graduate schooling	43 (24.7)	10.6	1.8		3.2	1.8		2.3	2.2	
College graduate	77 (44.3)	11.3	2.0		3.6	2.0		2.2	2.1	
High school graduate or equivalent	12 (6.9)	13.7	1.8		5.4	2.3		3.6	2.3	
Junior college graduate, or some college or technical school	30 (17.2)	11.8	2.5		5.5	2.2		3.2	2.1	
Unknown	12 (6.9)	9.5	1.7		3.2	2.3		3.5	2.1	
**Paternal education level (%)**				>0.05			>0.05			<0.001
Any post-graduate schooling	27 (15.5)	9.5	1.6		3.3	1.7		1.8	1.9	
College graduate	61 (35.1)	12.0	2.2		4.1	2.0		2.6	2.1	
High school graduate or equivalent	31 (17.8)	14.0	2.4		4.5	2.9		4.1	2.4	
Junior college graduate, or some college or technical school	40 (23.0)	10.7	1.5		3.8	1.7		2.0	2.0	
Unknown	15 (8.6)	8.3	1.8		3.2	2.3		3.1	2.1	
**Child's sex (%)**				>0.05			>0.05			> 0.05
Male	94 (54.0)	11.3	2.0		3.8	1.8		2.4	2.4	
Female	80 (46.0)	11.1	2.0		3.9	2.3		2.7	2.0	
**Collection season (%)**				>0.05			>0.05			> 0.05
winter	33 (19.0)	12.6	2.3		4.4	2.4		2.7	2.3	
spring	49 (28.2)	13.3	1.9		3.8	2.3		2.5	2.4	
summer	54 (31.0)	10.0	1.7		3.2	1.9		2.3	1.8	
fall	38 (21.8)	9.6	2.2		4.6	1.5		2.8	2.4	
**Primary home heating appliances (%)**				>0.05			>0.05			>0.05
Fireplace	1 (0.6)	9.7			1.1			1.0		
Forced air (furnace)	66 (37.9)	11.4	2.1		3.7	2.4		2.4	2.3	
Pellet stove	10 (5.7)	12.4	2.5		4.7	2.2		4.0	2.0	
Radiators (boiler)	42 (24.1)	10.5	1.8		3.7	1.6		2.1	2.1	
Wood stove	44 (25.3)	11.8	2.1		4.1	2.2		3.2	2.2	

Other	7 (4.0)	10.5	1.6		3.9	1.7		1.9	1.6	
Unknown	4 (2.3)	8.6	1.4		3.9	1.2		2.1	1.5	
**Primary home heating fuel (%)**				>0.05			>0.05			>0.05
Electricity	6 (3.4)	10.8	2.2		2.8	3.1		2.5	2.7	
Kerosene	1 (0.6)	15.5			13.7			2.7		
Natural Gas	5 (2.9)	12.9	1.6		4.1	1.2		3.1	2.4	
Oil	55 (31.6)	10.0	2.0		4.0	1.8		2.2	2.1	
Propane	48 (27.6)	11.9	2.0		3.2	2.2		2.3	2.2	
Wood	45 (25.9)	11.8	2.1		4.1	2.1		3.1	2.2	
Wood pellets	11 (6.3)	12.6	2.4		4.9	2.1		3.9	1.9	
Unknown	3 (1.7)	8.1	1.5		3.9	1.2		2.4	1.7	
**Fireplace exposure (yes/no) (%)**				>0.05			>0.05			>0.05
Yes	2 (1.1)	11.1	1.3		6.6			6.9	2.9	
No	171 (98.3)	11.2	2.0		3.8	2.1		2.5	2.2	
Unknown	1 (0.6)	13.0			3.3			2.9		
**Woodstove exposure (yes/no) (%)**				<0.05			>0.05			<0.005
Yes	30 (17.2)	14.6	2.2		4.9	2.3		4.0	2.4	
No	142 (81.6)	10.6	1.9		3.6	2.0		2.3	2.1	
Unknown	2 (1.1)	15.0	1.2		4.0	1.3		2.5	1.3	
**Exposure to tobacco smoke (yes/no) (%)**				>0.05			<0.0005			>0.05
Yes	20 (11.5)	14.3	2.4		6.8	2.3		3.0	2.1	
No	151 (86.8)	10.9	1.9		3.6	2.0		2.5	2.2	
Unknown	3 (1.7)	11.2	1.4		2.7	1.8		1.7	1.8	
**Maternal smoking history (ever/never) (%)**				<0.05			>0.05			>0.05
Yes	17 (9.8)	16.0	2.1		5.2	2.9		3.4	2.3	
No	145 (83.3)	10.9	2.0		3.7	1.9		2.4	2.2	
Unknown	12 (6.9)	9.3	2.0		3.5	2.4		3.3	2.1	
**Air filters use (yes/no) (%)**				>0.05			>0.05			>0.05
Yes	21 (12.1)	9.1	1.8		3.6	1.6		2.1	2.4	
No	152 (87.4)	11.5	2.0		3.9	2.1		2.6	2.2	
Unknown	1 (0.6)	13.0			3.3			2.9		
**Number of air filters at home (%)**				>0.05			>0.05			>0.05

0	152 (87.4)	11.5	2.0		3.9	2.1		2.6	2.2	
1	13 (7.5)	7.2	1.5		3.5	1.6		1.6	1.9	
2	4 (2.3)	19.6	2.4		4.6	1.7		4.7	2.7	
3	3 (1.7)	9.3	1.3		2.3	2.1		2.9	4.0	
5	1 (0.6)	8.3			5.1			1.4		
Unknown	1 (0.6)	13.0			3.3			2.9		
**Air filter in child's bedroom (yes/no) (%)**				>0.05			>0.05			>0.05
Yes	21 (12.1)	9.1	1.8		3.6	1.6		2.1	2.4	
No	152 (87.3)	11.5	2.0		3.9	2.1		2.6	2.2	
Unknown	1 (0.6)	13.0			3.3			2.9		

*P-value is derived by applying the overall ANOVA test to the log transformed exposure values

Table S3B. **Regression analyses: percentage change in $PM_{2.5}$, BC, and BrC concentrations for each type of exposure in households including covariates.**

	Type of Exposure											
	**Woodstove (yes/no)**	**95% CI**	**p-value**	**Hours woodstove used per day on average**	**95% CI**	**p-value**	**Woodstove primary heat source (yes/no)**	**95% CI**	**p-value**	**A single air filter in use (yes/no)**	**95% CI**	**p-value**
**Percentage change in PM2.5 levels per one unit change**												
Exposure	17.48	(3.1, 33.86)	<0.05	1.17	(0.33, 2.01)	<0.05	2.33	(-8.15, 14.01)	>0.05	-23.38	(-35.24, -9.34)	<0.005
BMI (kg/m²)	-4.37	(-7.77, -0.86)	<0.05	-3.88	(-7.3, -0.33)	<0.05	-4.63	(-8.11, -1.03)	<0.05	-4.23	(-7.58, -0.75)	<0.05
Hours of sleep per night on average	-0.98	(-1.61, -0.34)	<0.005	-0.91	(-1.54, -0.28)	<0.05	-0.95	(-1.61, -0.3)	<0.005	-0.96	(-1.59, -0.34)	<0.005
Season fall (yes/no)	-10.76	(-22.39, 2.62)	>0.05	-11.79	(-23.29, 1.44)	>0.05	-4.75	(-16.63, 8.81)	>0.05	-3.68	(-15.25, 9.48)	>0.05
Season winter (yes/no)	0.43	(-13.02, 15.96)	>0.05	1.66	(-11.5, 16.78)	>0.05	7.01	(-6.9, 23)	>0.05	10.84	(-3.19, 26.9)	>0.05
Season spring (yes/no)	5.63	(-6.53, 19.37)	>0.05	6.99	(-5.13, 20.67)	>0.05	8.97	(-4, 23.69)	>0.05	10.76	(-1.77, 24.89)	>0.05
**Percentage change in BC levels per one unit change**												
Exposure	18.92	(4.25, 35.65)	<0.05	1.61	(0.74, 2.5)	<0.005	13.32	(0.39, 27.92)	<0.05	-16.37	(-31.18, 1.62)	>0.05

Enrollment age (years)	-0.57	(-1.71, 0.59)	>0.05	-0.50	(-1.63, 0.64)	>0.05	-0.37	(-1.58, 0.86)	>0.05	-0.63	(-1.79, 0.53)	>0.05
Mother's education*	-18.60	(-47.64, 26.54)	>0.05	-19.84	(-47.98, 23.53)	>0.05	-23.31	(-51, 20.04)	>0.05	-18.89	(-48.03, 26.59)	>0.05
Father's education*	-21.18	(-48.23, 20.01)	>0.05	-22.60	(-48.73, 16.86)	>0.05	-25.76	(-51.56, 13.77)	>0.05	-19.93	(-47.71, 22.6)	>0.05
**Percentage change in BrC levels per one unit change**												
Exposure	22.27	(7.11, 39.58)	<0.005	0.97	(0.11, 1.84)	<0.05	3.62	(-8.14, 16.9)	>0.05	5.24	(-13.32, 27.77)	>0.05
BMI (kg/m^2)	-7.33	(-11, -3.5)	<0.005	-7.04	(-10.81, -3.1)	<0.005	-7.57	(-11.38, -3.6)	<0.005	-7.69	(-11.48, -3.74)	<0.005
Child's age (months)	-2.21	(-4.15, -0.24)	<0.05	-2.33	(-4.3, -0.33)	<0.05	-2.37	(-4.39, -0.32)	<0.05	-2.32	(-4.32, -0.28)	<0.05

**Table S4. Selected Characteristics of the Study population (participants with monitor wearing compliance ≥ 30%) and Participants Excluded (wearing compliance < 30%)**			
	**Study population, N=174**	**Excluded, N=84**	**P-value**
**PM2.5, GM (GSD)**	11.22 (2.0)	10.23 (2.24)	>0.05
**BC, GM (GSD)**	2.51 (2.24)	2.69 (2.34)	>0.05
**BrC, GM (GSD)**	3.47 (2.29)	3.31 (2.69)	>0.05
**Hours woodstove used per day on average, mean (SD)**	2.07 (5.69)	3.05 (6.77)	>0.05
**BMI (kg/m2), mean (SD)**	16.18 (1.53)	16.29 (1.38)	>0.05
**Child's age (months), mean (SD)**	45.94 (2.50)	46.12 (2.61)	>0.05
**Hours of sleep per night on average, mean (SD)**	32.49 (7.62)	33.35 (7.03)	>0.05
**Enrollment age (years), mean (SD)**	31.86 (4.89)	32.64 (4.82)	>0.05
**Ever smoker, (%)**			
Yes	17 (10.5)	9 (11.7)	
No	145 (89.5)	68 (88.3)	
**Air filter in use, (%)**			>0.05
Yes	21 (12.1)	9 (10.7)	
No	152 (87.9)	75 (89.3)	
**Number of air filters in use, (%)**			>0.05
0	152 (87.9)	75 (89.3)	
1	13 (7.5)	8 (9.5)	
2	4 (2.3)	1 (1.2)	
3	3 (1.7)	0 (0.0)	
5	1 (0.6)	0 (0.0)	
**Season, (%)**			<0.005
Fall	38 (21.8)	26 (31.0)	
Winter	33 (19.0)	28 (33.3)	
Spring	49 (28.2)	14 (16.7)	
Summer	54 (31.0)	16 (19.0)	
**Mother's education (%)**			>0.05
Less than 11th grade	0 (0.0)	1 (1.2)	
High school graduate or equivalent	12 (6.9)	5 (6.0)	
Junior college graduate, or some college or technical school	30 (17.2)	12 (14.3)	
College graduate	77 (44.3)	26 (31.0)	
Any post-graduate schooling	43 (24.7)	32 (38.1)	
**Father's education, (%)**			>0.05
Less than 11th grade	0 (0.0)	2 (2.4)	
High school graduate or equivalent	31 (17.8)	22 (26.2)	
Junior college graduate, or some college or technical school	40 (23.0)	17 (20.2)	
College graduate	61 (35.1)	21 (25.0)	
Any post-graduate schooling	27 (15.5)	13 (15.5)	

*The missing values are the omitted category in the table, they can be calculated by subtracting the non-missing categories from the totals. The observations with the missing covariates were excluded from the analyses. GM: Geometric Mean; GSD: Geometric Standard Deviation

Table S5. Percent of the nephelometer measurements less than the indicated $PM_{2.5}$ concentration (mg/m^3) descriptive statistic. The basis is the number of 1-minute average concentrations for each of the 211 participants with valid nephelometer files. Data corresponds to Figure 3.

Percent Less Than	Mean	SD	Median	10th%	25th%	75th%	90th%	Min	Max
0.5	1.6	2.1	0.9	<0.1	0.3	2.0	3.6	0.0	11.4
1	1.7	2.1	1.0	0.1	0.4	2.1	4.4	0.0	11.4
5	2.3	2.3	1.7	0.4	1.0	2.9	5.3	0.0	11.7
10	2.9	2.8	2.1	0.6	1.1	3.4	6.5	0.0	18.8
16	3.3	3.3	2.4	0.8	1.4	3.9	7.3	0.0	25.0
25	3.9	4.0	2.8	0.9	1.6	4.5	7.5	0.0	31.3
30	4.3	4.6	3.1	1.1	1.8	4.8	8.5	0.1	37.5
40	5.2	6.0	3.6	1.3	2.4	5.5	10.0	0.1	47.0
50	6.5	8.5	4.5	1.5	2.9	7.5	11.9	0.1	87.4
60	7.7	10.3	5.0	2.0	3.4	8.4	14.3	0.2	108.9
75	12.0	18.2	7.1	2.9	4.7	12.2	19.3	0.2	174.1
80	14.4	24.1	8.6	3.5	5.2	13.2	22.0	0.2	253.9
84	17.0	28.5	10.4	4.2	6.2	16.6	26.2	0.2	302.1
90	23.4	39.7	13.4	4.8	8.3	23.6	36.2	0.2	353.6
95	34.3	51.8	21.1	6.7	11.4	35.6	66.3	0.3	426.3
96	39.3	55.8	23.7	7.1	12.7	40.6	79.1	0.3	440.4
97	46.5	62.2	26.6	8.1	14.4	49.1	102.1	0.3	456.2
98	58.3	75.4	34.1	10.3	16.8	64.5	130.6	0.8	476.3
99	84.5	113.4	46.4	12.2	22.8	90.9	190.1	1.0	797.1
99.5	116.6	165.8	61.9	14.7	29.9	121.9	272.2	1.4	1305.5

Table S6. Distribution of nephelometer hourly average $PM_{2.5}$ concentration by hour of day for the 211 participants with valid nephelometer files. Data corresponds to Figure 4.

Hour of Day	Mean	SD	Median	25th%	75th%	Min	Max
0	11.5	24.6	5.2	3.3	10.4	0.1	224.7
1	10.4	21.8	4.9	3.1	8.4	0.1	171.4
2	10.0	21.4	4.5	3.0	8.2	0.1	180.4
3	9.5	20.3	4.3	2.9	8.2	0.1	184.3
4	9.1	20.3	4.2	2.8	7.6	0.1	181.1
5	9.2	21.7	4.2	2.8	7.6	0.1	215.3
6	9.7	17.7	4.8	3.2	8.5	0.2	138.7
7	11.2	19.0	5.8	3.8	10.6	0.2	198.5
8	11.6	13.4	7.3	4.3	13.3	0.2	102.7
9	13.9	24.4	8.3	4.6	13.2	0.2	264.3
10	11.8	15.8	7.7	4.8	12.6	0.2	188.8
11	10.5	9.8	7.7	4.8	12.2	0.2	71.6
12	10.1	9.2	7.9	4.5	11.9	0.2	75.6
13	10.1	11.5	7.1	4.1	11.7	0.1	125.6
14	10.0	14.1	7.0	4.3	10.8	0.2	161.5
15	9.2	10.9	6.4	4.4	10.5	0.4	104.9
16	10.2	10.1	6.9	4.6	12.1	0.1	75.3
17	14.2	23.5	8.0	5.1	13.9	0.2	272.4
18	17.9	31.9	9.0	5.5	17.0	0.2	330.5
19	15.0	19.2	8.7	5.2	16.4	0.2	160.4
20	14.2	23.4	7.9	4.7	14.8	0.2	282.1
21	13.8	28.1	7.1	4.2	13.3	0.2	351.8
22	14.0	33.8	6.3	3.8	11.6	0.1	379.1
23	13.1	32.8	6.0	3.4	11.1	0.1	398.7

Table S7. Sensitivity analyses: percentage change in $PM_{2.5}$, BC, and BrC concentrations for each type of exposure in households without air filters.

	Percent change per one unit change	95% CI	p-value
**$PM_{2.5}$**			
Any Woodstove Use	18.39	(3.09, 35.96)	<0.05
Hours of Woodstove use per day on average	1.55	(0.64, 2.46)	<0.005
Woodstove as a primary heating source	3.82	(-7.36, 16.34)	>0.05
**BC**			
Any Woodstove Use	18.92	(4.25, 35.65)	<0.05
Hours of Woodstove use per day on average	1.61	(0.74, 2.5)	<0.005
Woodstove as a primary heating source	13.32	(0.39, 27.92)	<0.05
**BrC**			
Any Woodstove Use	25.20	(8.91, 43.93)	<0.005
Hours of Woodstove use per day on average	1.18	(0.24, 2.12)	<0.05
Woodstove as a primary heating source	4.56	(-8.23, 19.12)	>0.05

*Models for $PM_{2.5}$ were adjusted for child BMI (kg/m^2), child age, hours of sleep per night on average, and season of collection. Models for BC adjusted for maternal age at enrollment, mother and father highest level of education attained. Models for BrC adjusted for child's age and BMI (see text).

FIGURE S1. Halter style vest that the study participants wore to carry the MicroPEM with minimal burden.

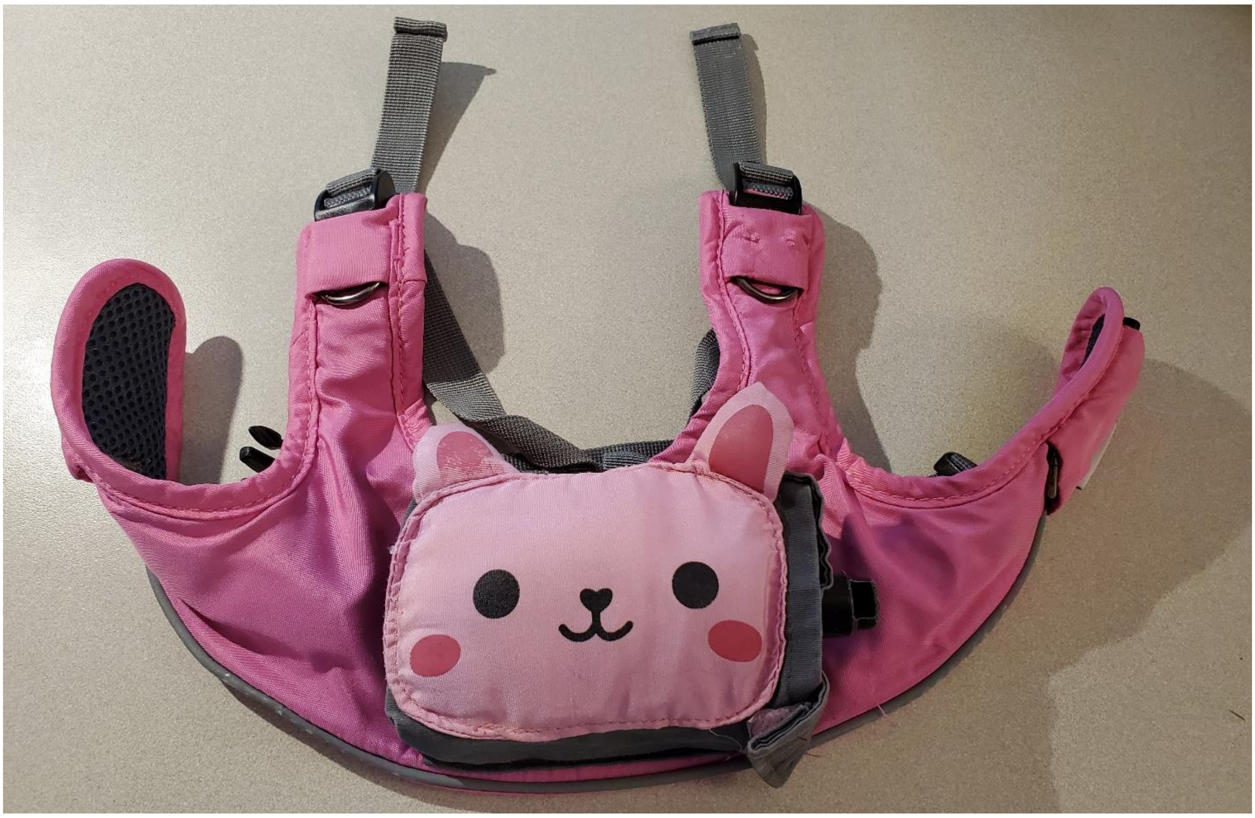

Supplement: Supplementary file 1 [file ijerph-18-12109-s001.zip › ijerph-1439173-supplementary.pdf]
